# Comprehensive analysis of coding and non-coding RNA transcriptomes related to hypoxic adaptation in Tibetan chickens

**DOI:** 10.1186/s40104-021-00582-2

**Published:** 2021-05-03

**Authors:** Ying Zhang, Woyu Su, Bo Zhang, Yao Ling, Woo Kyun Kim, Hao Zhang

**Affiliations:** 1 National Engineering Laboratory for Animal Breeding, Plateau Animal Genetic Resources Center, China Agricultural University, No. 2 Yuanmingyuan West Rd, Haidian District, Beijing, 100193 China; 2 Department of Poultry Science, University of Georgia, 303 Poultry Science Building, Athens, GA 30602 USA

**Keywords:** ceRNA, Hypoxic adaptation, Non-coding RNA, Tibetan chicken, Transcriptome

## Abstract

**Background:**

Tibetan chickens, a unique native breed in the Qinghai-Tibet Plateau of China, possess a suite of adaptive features that enable them to tolerate the high-altitude hypoxic environment. Increasing evidence suggests that long non-coding RNAs (lncRNAs) and microRNAs (miRNAs) play roles in the hypoxic adaptation of high-altitude animals, although their exact involvement remains unclear.

**Results:**

This study aimed to elucidate the global landscape of mRNAs, lncRNAs, and miRNAs using transcriptome sequencing to construct a regulatory network of competing endogenous RNAs (ceRNAs) and thus provide insights into the hypoxic adaptation of Tibetan chicken embryos. In total, 354 differentially expressed genes (DE genes), 389 differentially expressed lncRNAs (DE lncRNAs), and 73 differentially expressed miRNAs (DE miRNAs) were identified between Tibetan chickens (TC) and control Chahua chickens (CH). GO and KEGG enrichment analysis revealed that several important DE miRNAs and their target DE lncRNAs and DE genes are involved in angiogenesis (including blood vessel development and blood circulation) and energy metabolism (including glucose, carbohydrate, and lipid metabolism). The ceRNA network was then constructed with the predicted DE gene-DE miRNA-DE lncRNA interactions, which further revealed the regulatory roles of these differentially expressed RNAs during hypoxic adaptation of Tibetan chickens.

**Conclusions:**

Analysis of transcriptomic data revealed several key candidate ceRNAs that may play high-priority roles in the hypoxic adaptation of Tibetan chickens by regulating angiogenesis and energy metabolism. These results provide insights into the molecular mechanisms of hypoxic adaptation regulatory networks from the perspective of coding and non-coding RNAs.

**Supplementary Information:**

The online version contains supplementary material available at 10.1186/s40104-021-00582-2.

## Background

MicroRNAs (miRNAs) are small non-coding RNA molecules that inhibit gene expression by binding to specific mRNAs. LncRNAs are another type of non-coding RNA, with a length of more than 200 nucleotides (nt), that regulate gene expression through a number of mechanisms, including epigenetic regulation (genetic imprinting and chromatin remodeling), transcriptional regulation (transcription interference), post-transcriptional regulation (splicing), and so on [1]. Both of these non-coding RNAs play different roles in various aspects of cellular function, including the regulation of hypoxia-related genes. Through their activity on target transcripts, some critical miRNAs regulate angiogenesis, the cell cycle, as well as lactate transport under hypoxic conditions [2–4]. *HIF-1* and *VEGF* are regulated by miR-17-92, miR-20b, and miR-199a, which play roles in tumor development, cancer cell proliferation, and cardiac myocytes [5–7]. Various studies have shown that lncRNAs can directly bind to *HIF-1* and activate its expression, in turn modulating hypoxic responses similar to the suppression of cancer cell adaptation to hypoxia, which provides important insights into how tumor cells sense and adapt to hypoxic stress [8–10]. Although there have been various studies on genes involved in high-altitude adaptation in humans and animals, the regulatory mechanism of non-coding RNAs involved in hypoxic adaptation remains largely unknown.

Tibetan chickens, which live in Tibetan highlands and have undergone long-term natural selection, exhibit stable genetic adaptation to high-altitude environments, characterized by high hatchability under hypoxic incubation when compared to lowland chickens [11, 12]. This indicates that Tibetan chickens have special physiological and genetic mechanisms enabling them to adapt to extreme high-elevation environments, and ensuring their normal hatching, reproduction, and growth in plateau environments [13–15]. Previous studies using genomic, transcriptomic, and proteomic profiles have identified several functional genes related to hypoxic adaptation in Tibetan chickens [16–18]. However, the mechanisms involved in the regulation of hypoxic adaptation by non-coding RNA are unclear.

Chorioallantoic membrane (CAM), which functions as both a respiratory and circulatory organ for the chicken embryo, contains numerous blood vessels. Tibetan chicken embryos have blunted responses to hypoxia on CAM angiogenesis, which might benefit blood flow and oxygen transportation during hypoxic incubation [18]. The characteristics of hypoxic adaptation may involve regulation of expressed mRNAs and non-coding RNAs; however, this remains unclear. In this study, full transcriptome sequencing (RNA-seq) of chicken embryos was performed to explore the regulation of non-coding RNAs and candidate genes responsible for hypoxic adaptation traits in Tibetan chickens as compared to the Chahua chickens. Integration of three types of RNAs (mRNAs, lncRNAs and miRNAs) revealed competing endogenous RNA (ceRNA) interactive network and potential regulatory mechanism to explain hypoxic adaptation in Tibetan chickens. These findings will help us further understand the molecular mechanism of adaptability to hypoxic conditions in Tibetan chickens from a non-coding RNA perspective.

## Methods

### Sample collection and preparation

The experiments and animal care protocol were approved by the animal welfare committee of the State Key Laboratory for Agro-Biotechnology of the China Agricultural University (approval number, XK257), and all methods were performed in accordance with the relevant guidelines and regulations.

We collected eggs from Tibetan chickens (TCs) and Chahua chickens (CHs) from the Experimental Station for Poultry Genetic Resources and Breeding, China Agricultural University (CAU). The two groups of eggs were incubated in a hypoxic incubator (13% ± 0.2% O_2_) and the temperature and humidity of the incubator were 37.8 °C and 60%, respectively. Eighteen CAM samples from Tibetan chickens (*n* = 9) and Chahua chickens (*n* = 9) were collected from female embryos at day 11 of incubation under hypoxic condition, immediately frozen in liquid nitrogen, and stored at − 80 °C for RNA extraction and sequencing.

### RNA extraction, library construction, and sequencing

Total RNA was extracted from the CAM samples using an RNA pure Tissue Kit (Tiangen Biotech Co. Ltd., Beijing, China) according to the manufacturer’s protocol. Six RNA-seq libraries (TC1, TC2, TC3, CH1, CH2, and CH3) were constructed, three biological duplicates were prepared, and each sample consisted of a mix from three individuals of the same breed.

For mRNA and lncRNA sequencing, 1.5 μg of total RNA was used as input material for rRNA removal using the Ribo-Zero rRNA Removal Kit (Epicentre, Madison, WI, USA). Sequencing libraries were generated using the NEBNext^R^ Ultra™ Directional RNA Library Prep Kit for Illumina^R^ (NEB, USA) following the manufacturer’s instructions, and 150 paired-end reads were generated. For small RNA sequencing, 1.5 μg of total RNA per sample was used to ligate the 3′ SR and 5′ SR Adaptor. Reverse transcription synthetic first chain and PCR amplification were carried out, PAGE gel was used to screen the electrophoresis fragment, then rubber cutting and recycling. The PCR products were purified (AMPure XP system) and library quality was assessed. Clustering of the index-coded samples was performed using TruSeq PE Cluster Kit v4-cBot-HS (Illumina) according to the manufacturer’s instructions, and 50-nt single-end reads were generated.

### Read mapping and transcriptome assembly

For mRNA and lncRNA data, using in-house Perl scripts, raw 150 bp paired-end reads were filtered by removing adapters and reads containing poly-N and low-quality sequences to obtain clean reads. Clean reads were aligned to the chicken reference genome Gallus-gallus-6.0 (GRCg6a-96.Gallus-gallus. GRCg6a-96.genome.fa) using the HISAT2 software [19]. The transcriptome was assembled using StringTie and annotated using the gffcompare program. For miRNA data, the 3′ linker sequences were removed from the raw 50-nt single-end reads, which were then filtered by removing reads with ≥10% base N and shorter than 18 or longer than 30 nucleotides to obtain clean reads. The clean reads were aligned with the Silva, GtRNAdb, Rfam, and Repbase databases to filter other non-coding RNAs, such as ribosomal RNA (rRNA), transfer RNA (tRNA), small nuclear RNA (snRNA), small nucleolar RNA (snoRNA), and repeating sequences to obtain unannotated reads containing miRNAs. Unannotated reads were mapped to the reference genome (*Gallus-gallus*. GRCg6a-96) using the Bowtie software [20]. The Q20, Q30, and GC content of the clean data were calculated for all RNAs.

### Identification of lncRNAs and prediction of their target genes

The unknown transcripts were screened as putative lncRNAs or protein-coding RNAs. The screening methods were used according to previous reports [21, 22], which briefly included length ≥ 200 bp, exon number ≥ 2, and FPKM ≥0.1, using tools of CPC2 (coding potential calculator), CNCI (coding-non-coding index), CPAT (coding potential assessment tool), and pfam protein domain analysis tools [23–26]. The transcripts were aligned with lncRNA databases (lncRNAdb and NONCODE) to identify known lncRNAs. Integration of following two methods to predict target genes of the lncRNAs: (1) lncRNA regulation of the expression of its neighboring genes, which is based on the distance (within 100 kb) between lncRNA and potential target genes in the genome, and (2) correlations between lncRNA and potential target gene expression in all samples that were significantly positive.

### Identification of miRNAs and prediction of their target genes

Known miRNAs were identified by mapping the matched mature miRNAs in miRBase (v22) to the reference genome. Novel miRNAs were predicted using miRDeep2 from the unannotated reads [27]. Target genes of miRNAs were predicted by miRanda and TargetScan based on the miRNA and gene sequence information [28, 29].

### RNA quantification and differential expression analysis

Expression level of mRNAs and lncRNAs were quantified based on fragments per kilobase of transcript per million fragments mapped (FPKM) using the StringTie software [30]. Expression levels of miRNAs were quantified and normalized using the TPM algorithm (TPM = Readcount × 1,000,000/mapped reads) [31]. The DESeq2 software package was used to detect differentially expressed lncRNAs (DE lncRNAs), differentially expressed genes (DE genes), and differentially expressed miRNAs (DE miRNAs) with criteria of log_2_| fold change| > 1 and *P* < 0.05 [32].

### GO and KEGG enrichment analysis of differentially expressed RNA

Gene Ontology (GO) enrichment analysis of the DE genes was performed using the GOseq R package based on Wallenius’ non-central hypergeometric distribution. Kyoto Encyclopedia of Genes and Genomes (KEGG) is a database resource for understanding high-level gene functions and utilities of a biological system, such as the cell, organism, and ecosystem, using molecular-level information, especially large-scale molecular datasets generated by genome sequencing and other high-throughput experimental technologies (http://www.genome.jp/kegg/). We used the KOBAS software to test the statistical significance of DE gene enrichment in KEGG pathways.

### Construction of the ceRNA network

Competitive endogenous RNAs (ceRNAs) are novel transcriptional regulators that can modulate the expression of transcripts through competitive binding. The interactions of miRNA-mRNA and miRNA-lncRNA was predicted using TargetScan and miRanda, and the competitive pairs of mRNA-lncRNA were identified using hypergeometric test and Pearson correlation coefficient. Based on the sharing of miRNA binding sites and competitive pairs between lncRNAs and mRNAs, ceRNA network was constructed as follows: *P* < 0.01 and FDR < 0.01 in hypergeometric test [33–35]. Visualization of ceRNA using Cytoscape software (http://cytoscape.org/).

### Expression analysis of DE lncRNAs, DE miRNAs, and DE genes using qRT-PCR

To confirm the differentially expressed RNAs identified through RNA-seq, quantitative real-time PCR (qRT-PCR) was carried out for six DE lncRNAs, six DE miRNAs, and seven DE genes. We used the PrimeScript RT reagent kit with gDNA (Tiangen Biotech Co. Ltd., Beijing, China) to convert total RNA to cDNA, using random hexamers for mRNA and lncRNAs, and stem-loop RT primers for miRNAs. The qPCR was performed using the SYBR Green PCR Kit (Tiangen Biotech Co. Ltd., Beijing, China) according to the manufacturer’s instructions. The internal controls included *GAPDH* (glyceraldehyde-3-phosphate dehydrogenase) for lncRNAs, chicken 5S rRNA for miRNAs, and *HPRT* (hypoxanthine guanine phosphoribosyl transferase) for mRNAs. All primers used in qRT-PCR are shown in Additional file 1: Table S1. The BioRad CFX96 (Bio-Rad, CA, USA) was used to perform qRT-PCR with the SYBR Green PCR Master Mix (Tiangen Biotech Co. Ltd.). Each qRT-PCR experiment was performed in triplicate, relative RNA expression levels were calculated using the 2^−^^ΔΔCT^ method, and ANOVA testing via SPSS 25.0 was applied. The results are expressed as mean ± SE, and *P* ≤ 0.05 was considered statistically significant. Software and associated parameters used are shown in Additional file 2: Table S2.

## Results

### Overview of RNA transcriptomic profiles of chicken CAM tissues

**Fig. 1:**
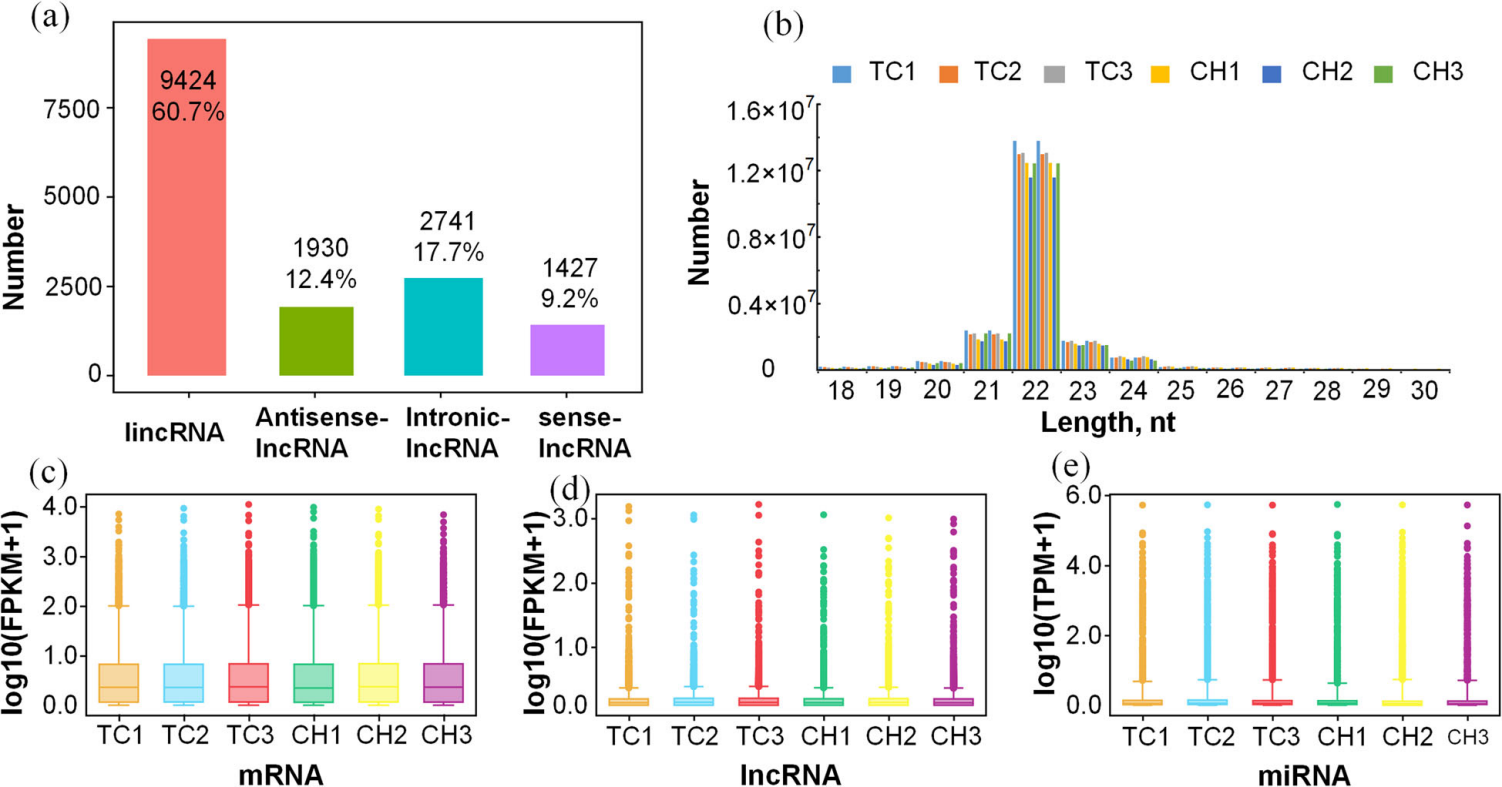
Comparison of the features of RNAs in the chorioallantoic membrane of chicken embryos. (**a**) Statistics of long non-coding RNA, including intergenic non-coding RNAs (lincRNAs), anti-sense lncRNAs, intronic lncRNAs, and sense lncRNAs; (**b**) Length distribution of 1439 miRNAs; (**c**) Distribution of mRNA expression values (FPKM) of six samples; (**d**) Distribution of lncRNA expression values (FPKM) of six samples; (**e**) Distribution of miRNA expression values (TPM) of six samples. The ordinate shows the log_10_(FPKM + 1) (**c**, **d**) and the log_10_(TPM + 1) (**e**) of each RNA from the six sets of RNA-seq data; the middle line in the box represents the median of FPKM/TPM. TC, Tibetan chicken; CH, Chahua chicken

After low-quality reads were removed, 110.3–114.1 M paired-end reads (2 × 150 bp in length) for the mRNAs and lncRNAs, and 23.8–29.2 M single-end reads (50 bp in length) for the miRNAs, were obtained from the CAM of Tibetan and Chahua chicken samples (Additional file 3: Table S3). The majority of the lncRNAs (60.7%) were intergenic long-stranded non-coding RNA (lincRNA), followed by intronic-lncRNA (17.7%), antisense-lncRNA (12.4%), and 9.2% sense-lncRNA (Fig. 1a). The length of mRNAs ranged from 51 to 30,0721 bp, while that of lncRNAs ranged from 202 to 223,626 bp; the majority of mRNAs and lncRNAs were 201–400 bp and > 3000 bp long, respectively (Additional file 4: Fig. S1). In total, 20,912 expressed genes, 15,523 lncRNAs (3194 known), and 1439 miRNAs were identified in the CAM tissues of Tibetan and Chahua chickens. The 1439 miRNAs included 471 known and 968 novel miRNAs, and most of the miRNA reads were between 20 and 24 nt in length (Fig. 1b, Additional file 5: Table S4). The values of FPKM of mRNA/lncRNA and TPM of miRNA distribution were similar among the six samples (Fig. 1c, d, e).

### Differential expression analysis of RNAs between TC and CH

**Fig. 2:**
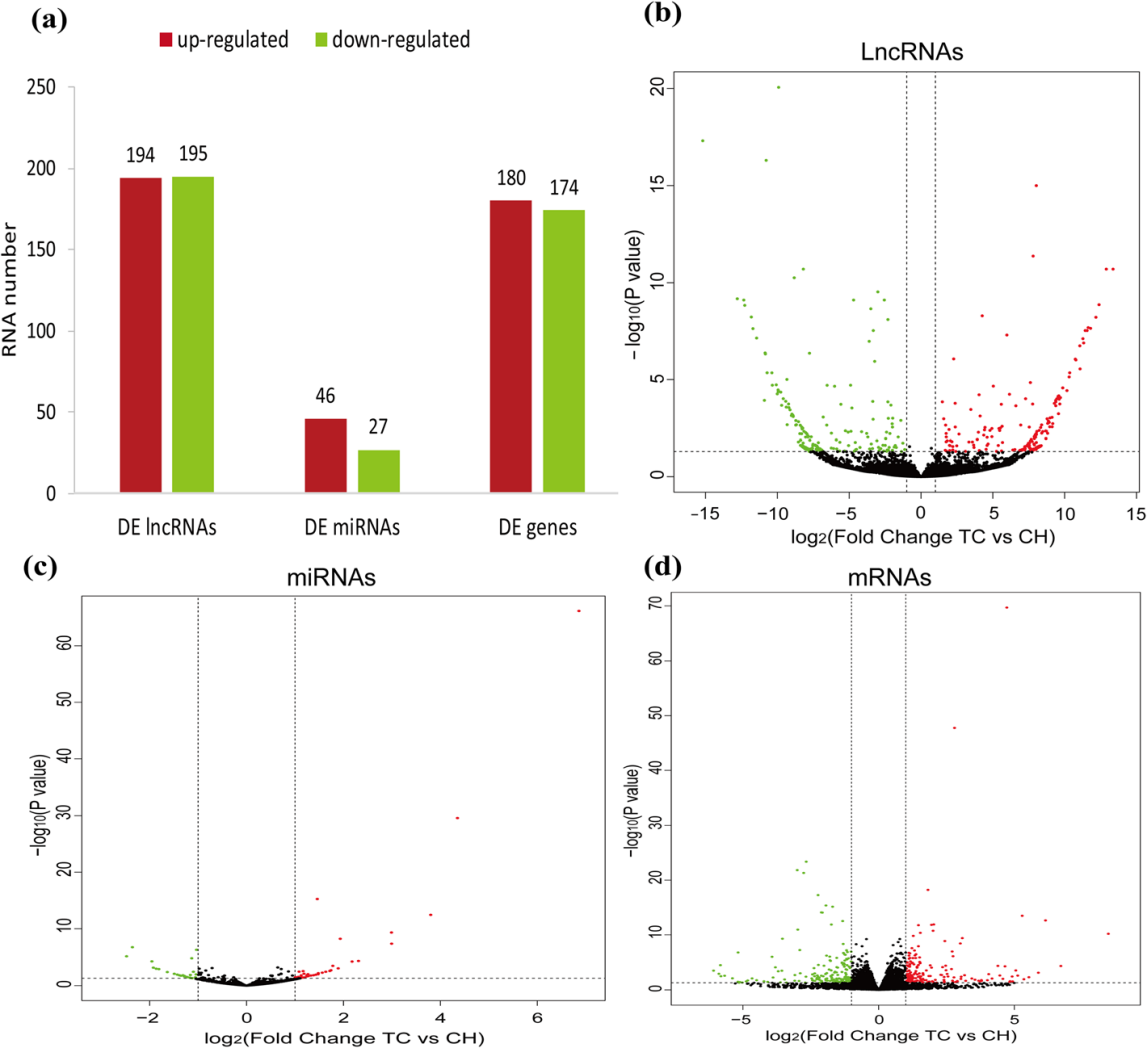
Identification of differentially expressed (DE) lncRNAs, DE miRNAs, and DE genes between Tibetan (TC) and Chahua chicken (CH). Number of DE lncRNAs, DE miRNAs, and DE genes (**a**); Volcano plots displaying DE lncRNAs (**b**), DE miRNAs (**c**), and DE genes (**d**) between Tibetan and Chahua chicken. Upregulated and downregulated genes are shown in red and green, respectively. Black dots represent genes with similar expression levels

We found 389 DE lncRNAs, 73 DE miRNAs, and 354 DE genes between the TC and CH groups, among which 194 DE lncRNAs, 46 DE miRNAs, and 180 DE genes were upregulated, whereas 195 DE lncRNAs, 27 DE miRNAs, and 174 DE genes were downregulated in the Tibetan chickens compared to Chahua chickens (Fig. 2, Additional file 6: Table S5). The DE lncRNAs, DE miRNAs, and DE genes were classified into two categories for the six samples, in which the expressed quantity of the DE lncRNAs, DE miRNAs, and DE genes had good repeatability within groups respectively (Additional file 4: Fig. S2).

### Functional analysis of differentially expressed non-coding RNAs (DE lncRNAs and DE miRNAs)

**Fig. 3:**
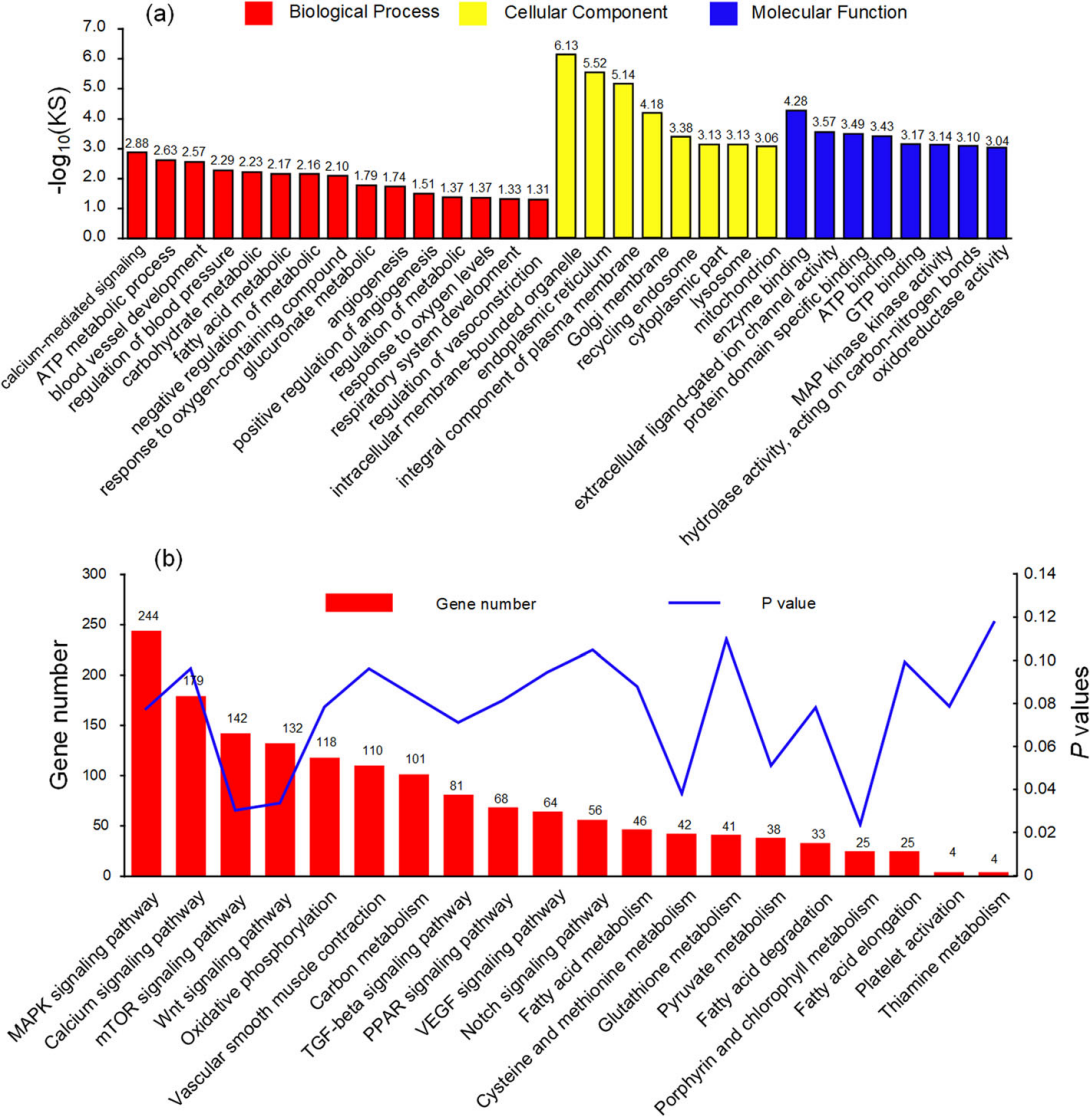
GO and KEGG enrichment analysis of differentially expressed (DE) lncRNAs. (**a**) GO enrichment analysis of DE lncRNAs between Tibetan (TCs) and Chahua chickens (CHs). KS represents the statistical significance of enriching the GO term, the smaller the KS value mean the more significant the enrichment; (**b**) KEGG enrichment analysis of DE lncRNAs between Tibetan and Chahua chickens

For 389 DE lncRNAs, we predicted 722 *cis*- and *trans*-target genes that were mainly involved in the GO terms of angiogenesis, blood vessel development, regulation of vasoconstriction, regulation of blood pressure, response to oxygen levels, ATP metabolic process, fatty acid metabolic process, glucuronate metabolic process, enzyme binding, and ATP binding (Fig. 3a, Additional file 7: Table S6a). The KEGG analysis revealed the involvement of target genes in carbon metabolism, fatty acid metabolism, vascular smooth muscle contraction, and the Wnt, mTOR, MAPK, VEGF, and calcium signaling pathways (Fig. 3b, Additional file 7: Table S6b).

**Fig. 4:**
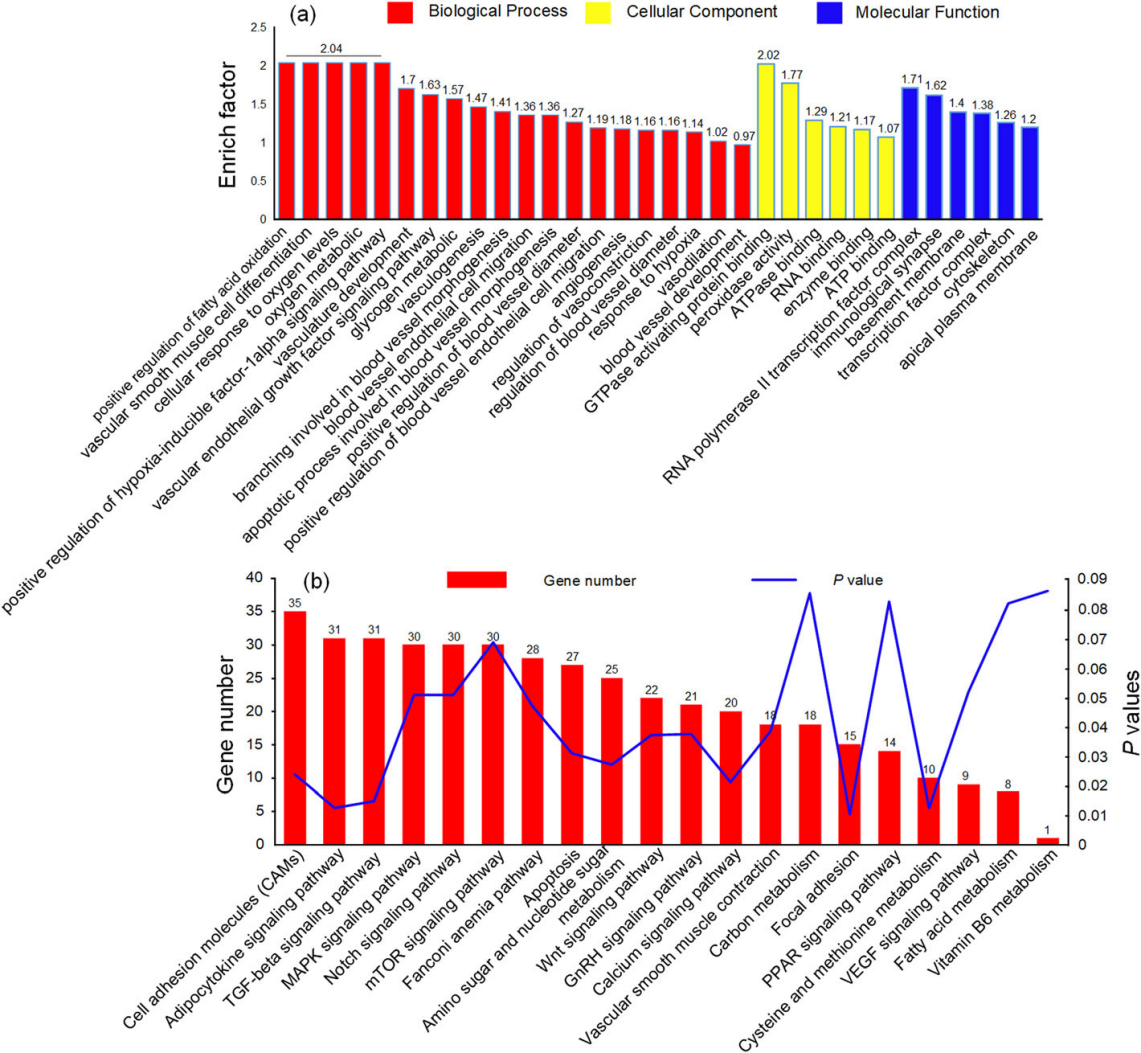
GO and KEGG enrichment analysis of differentially expressed (DE) miRNAs. (**a**) GO enrichment analysis of DE miRNAs between Tibetan (TCs) and Chahua chickens (CHs). The enrichment factor is the ratio of DE miRNA numbers annotated in the GO term to the total gene numbers annotated in the same GO term. (**b**) KEGG enrichment analysis of DE miRNAs between Tibetan and Chahua chickens

A total of 1766 target genes were predicted for 73 DE miRNAs. The target genes were mainly enriched in the GO terms of vasculogenesis, positive regulation of fatty acid oxidation, glycogen metabolic process, angiogenesis, vasculature development, response to hypoxia, oxygen metabolic process, vasodilation, ATP binding, and enzyme binding (Fig. 4a, Additional file 8: Table S7a). Notably, the enriched KEGG pathways were cysteine and methionine metabolism, amino sugar and nucleotide sugar metabolism, apoptosis, vascular smooth muscle contraction, fatty acid metabolism, carbon metabolism, vitamin B_6_ metabolism, and the MAPK, Notch, VEGF, mTOR, and calcium signaling pathways (Fig. 4b, Additional file 8: Table S7b).

### Comparative parsing of lncRNAs and mRNAs and functional analysis of DE genes

**Fig. 5:**
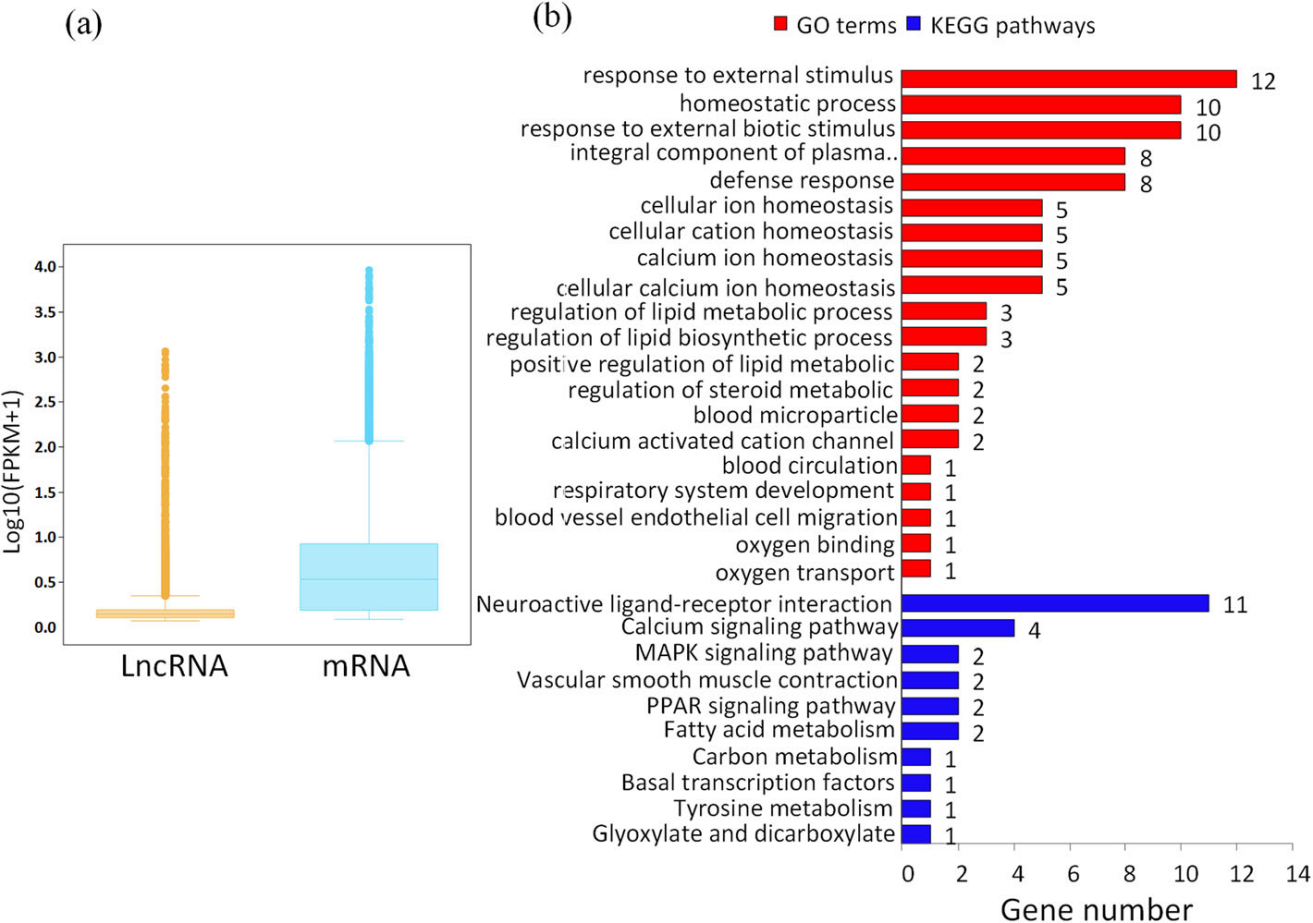
Comparative parsing of lncRNAs and mRNAs and functional analysis of differentially expressed (DE) genes. (**a**) A comparison of expression between lncRNAs and mRNAs. The box chart statistics from top to bottom are the maximum value, upper quartile, middle value, lower quartile, and the minimum value; (**b**) GO and KEGG enrichment analysis of DE genes between Tibetan (TC) and Chahua chicken (CH)

To compare the exon number, open reading frame (ORF), and expression levels of lncRNA and mRNA, we needed to consider the differences in structure and sequence. The number of exons corresponding to lncRNAs was mainly less than 8, whereas the number of exons in mRNAs was relatively large, ranging from 1 to > 30 (Additional file 4: Fig. S3a, b). The majority of lncRNA ORFs were mainly between 50 and 250 nt long, while the length of the ORFs of mRNAs was mainly between 100 and 1,000 nt (Additional file 4: Fig. S3c, d). Expressed mRNAs and lncRNAs were located on all chromosomes of the chicken genome (Additional file 4: Fig. S4). The average and maximum values of mRNA expression were higher compared to those of lncRNA expression (Fig. 5a).

The 354 DE genes were mainly enriched in GO terms of defense response, homeostatic process, regulation of lipid metabolic process, glucose homeostasis, blood microparticle, oxygen transport, blood vessel endothelial cell migration, blood circulation, and respiratory system development. Enriched KEGG pathways included fatty acid metabolism, vascular smooth muscle contraction, as well as the calcium, PPAR and MAPK signaling pathways (Fig. 5b, Additional file 9: Table S8). Twelve DE genes (*ACTC1*, *KCNMB4*, *NCS1*, *NGFR*, *ADAM8*, *CASQ2*, *IRF4*, *PTPRZ1*, *CALML3*, *ERBB4*, *ARR3*, and *NTSR1*) were mainly associated with angiogenesis, blood circulation, hematopoiesis, as well as calcium and MAPK signaling pathways. Fifteen DE genes (*SSTR5*, *NR1H4*, *HTR2C*, *APOA1*, *KCNB1*, *P3H2*, *CHST8*, *LYZ*, *HAO2*, *ACER1*, *ACSBG1*, *ELOVL2*, *ELOVL3*, *GBE*, and *NOX3*) were related to glucose and carbohydrate metabolism, fatty acid metabolism, and lactate oxidation (Table 1).

**Table 1:** Potential key differentially expressed mRNAs (DE genes), their targeted differentially expressed miRNAs (DE miRNAs)/differentially expressed lncRNAs (DE lncRNAs), and their functions related to hypoxic adaption in the Tibetan chicken

DE genes	Log_2_FC (TC/CH)	*P*-value	Targeted DE miRNAs	Targeted DE lncRNAs	Functional description
Angiogenesis					
ACTC1	−3.4644	0.0122	gga-miR-6606-5p	MSTRG.19949.13, MSTRG.128588.3, MSTRG.73129.28, MSTRG.32082.2	Blood circulation
KCNMB4	2.3558	0.0001	gga-miR-6606-5p	MSTRG.29252.7	Vascular smooth muscle contraction
NCS1	−1.0050	0.0197	gga-miR-6606-5p, novel_miR_815	MSTRG.33345.5, MSTRG.140060.1, MSTRG.18943.13	Calcium ion transport
NGFR	1.5519	0.0036	novel_miR_676	MSTRG.118362.24, MSTRG.12318.1, MSTRG.25780.4, MSTRG.128839.3, MSTRG.115756.46, MSTRG.41646.22, MSTRG.80622.27, MSTRG.44286.2, MSTRG.41646.23	Blood vessel morphogenesis, blood vessel development, vasculature development, angiogenesis
ADAM8	−1.3727	0.0470			Response to hypoxia, blood vessel development, vasculature development, angiogenesis
CASQ2	−1.0736	0.0315	novel_miR_589, novel_miR_676	MSTRG.73129.28, MSTRG.18943.3	Blood circulation
IRF4	−1.8880	0.0201			Hemopoiesis
PTPRZ1	1.3653	0.0000			Hemopoiesis, hematopoietic progenitor cell differentiation
CALML3	−1.1958	0.0015			Vascular smooth muscle contraction
ERBB4	−1.2949	0.0102	novel_miR_676, novel_miR_587	MSTRG.147643.141, MSTRG.128534.3, MSTRG.44329.1, MSTRG.29590.15, MSTRG.79818.8, MSTRG.135465.5, MSTRG.30620.3	Calcium and MAPK signaling pathways
ARR3	1.0513	0.0013			MAPK signaling pathway
NTSR1	−1.5401	0.0140	novel_miR_669, novel_miR_676, novel_miR_567	MSTRG.63187.27, MSTRG.71117.21, MSTRG.133793.2, MSTRG.29590.15, MSTRG.52146.15, MSTRG.72741.32, MSTRG.148631.1	Regulation of respiratory gaseous exchange, Calcium signaling pathway
Energy metabolism					
SSTR5	−3.8881	0.0054	novel_miR_819	MSTRG.25881.3	Glucose homeostasis
NR1H4	−1.1224	0.0179	novel_miR_676	MSTRG.99917.37, MSTRG.64717.15, MSTRG.99917.39, MSTRG.99917.35	Glucose homeostasis, response to oxygen-containing compound
HTR2C	−1.3476	0.0000			Regulation of lipid metabolic process
APOA1	1.1034	0.0068			Regulation of lipid metabolic process
KCNB1	−1.1800	0.0000			Glucose homeostasis, response to oxygen-containing compound
P3H2	−1.0044	0.0135			Carbohydrate binding
CHST8	−1.9553	0.0013	novel_miR_693, novel_miR_775		Carbohydrate metabolic process
LYZ	−1.0365	0.0050			Carbohydrate metabolic process
HAO2	−1.5483	0.0085	novel_miR_669		Carbon metabolism, lactate oxidation
ACER1	1.5111	0.0032			Regulation of lipid metabolic process
ACSBG1	1.1154	0.0000			Fatty acid metabolism
ELOVL2	−5.1386	0.0480			Fatty acid metabolism
ELOVL3	2.0872	0.0146	novel_miR_867		Fatty acid metabolism
GBE	−1.0082	0.0072	novel_miR_85	MSTRG.128588.4	Oxygen transport, oxygen binding, reactive oxygen species metabolic process
NOX3	3.0828	0.0000			Reactive oxygen species metabolic process

### Construction of the ceRNA network

**Fig. 6:**
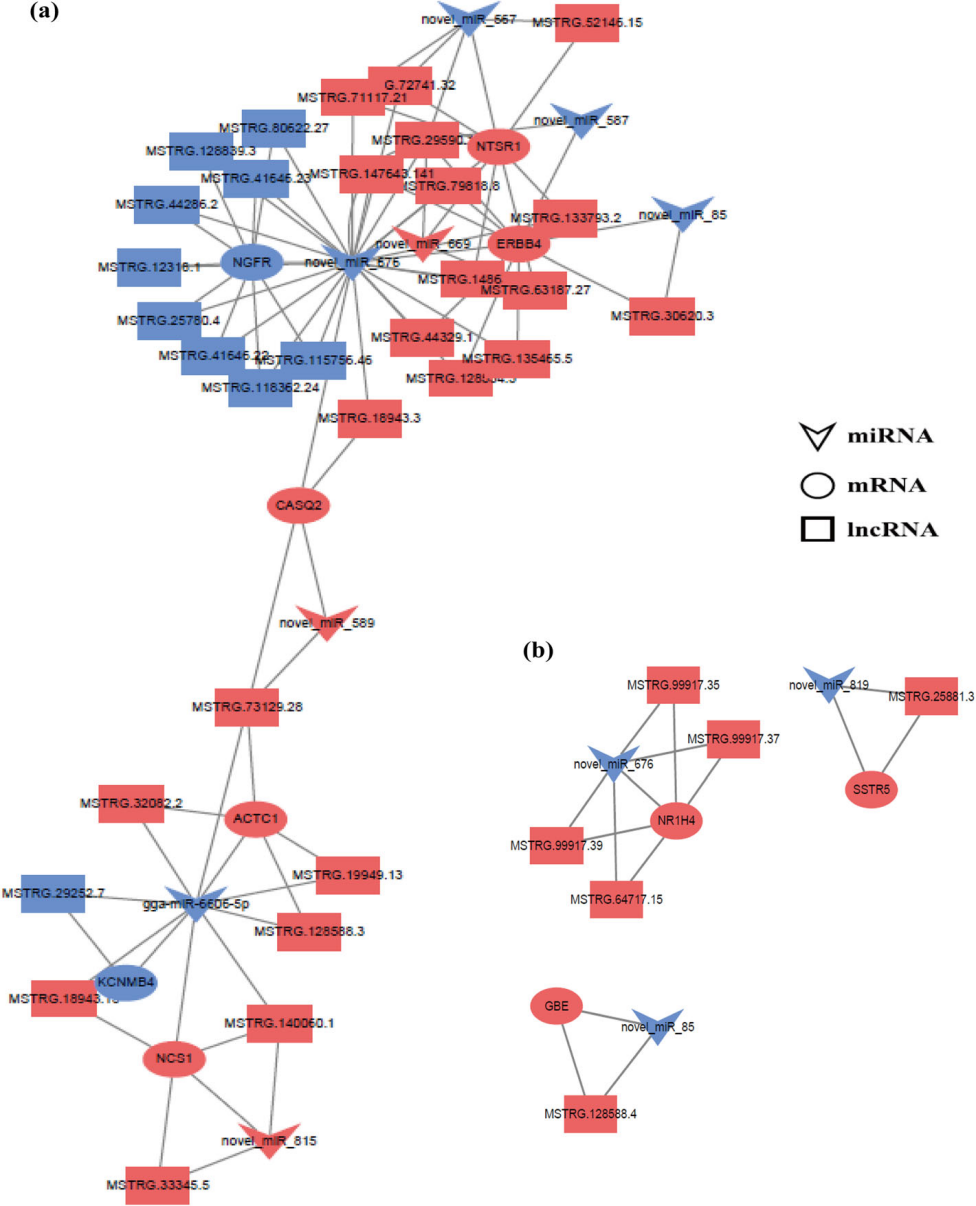
Mini-ceRNA network constructed with 10 differentially expressed (DE) genes and their 37 target DE lncRNAs and 9 target DE miRNAs, involved in angiogenesis (**a**) and energy metabolism (**b**). Red color represents the up-regulated and blue color represents down-regulated levels

We constructed a ceRNA network that yielded 529 pairs of candidate ceRNAs (lncRNA-miRNA-mRNA) from 162 DE lncRNAs and 108 DE genes through 25 miRNA target-mediated relationships (Additional file 4: Fig. S5). Considering that the network contains enormous information, and each relationship cannot be displayed in the figure, we constructed a mini-ceRNA (DE lncRNA-DE miRNA-DE mRNA) network of important RNAs. We identified 10 known mRNAs that were related to hypoxic adaptation and involved in angiogenesis and blood circulation (*NGFR*, *ACTC1*, *CASQ2*, *ERBB4*, *KCNMB4*, *NCS1*, and *NTSR1*) (Fig. 6a), as well as energy metabolism (*SSTR5*, *NR1H4*, and *GBE*) (Fig. 6b). A total of 39 lncRNA-miRNA-mRNA interactions were identified in the mini-ceRNA network constructed with these 10 DE genes and their targeted 37 DE lncRNAs and 9 DE miRNAs (Table 1, Additional file 10: Table S9).

### Expression levels of DE lncRNAs, DE miRNAs, and DE genes using qRT-PCR

**Fig. 7:**
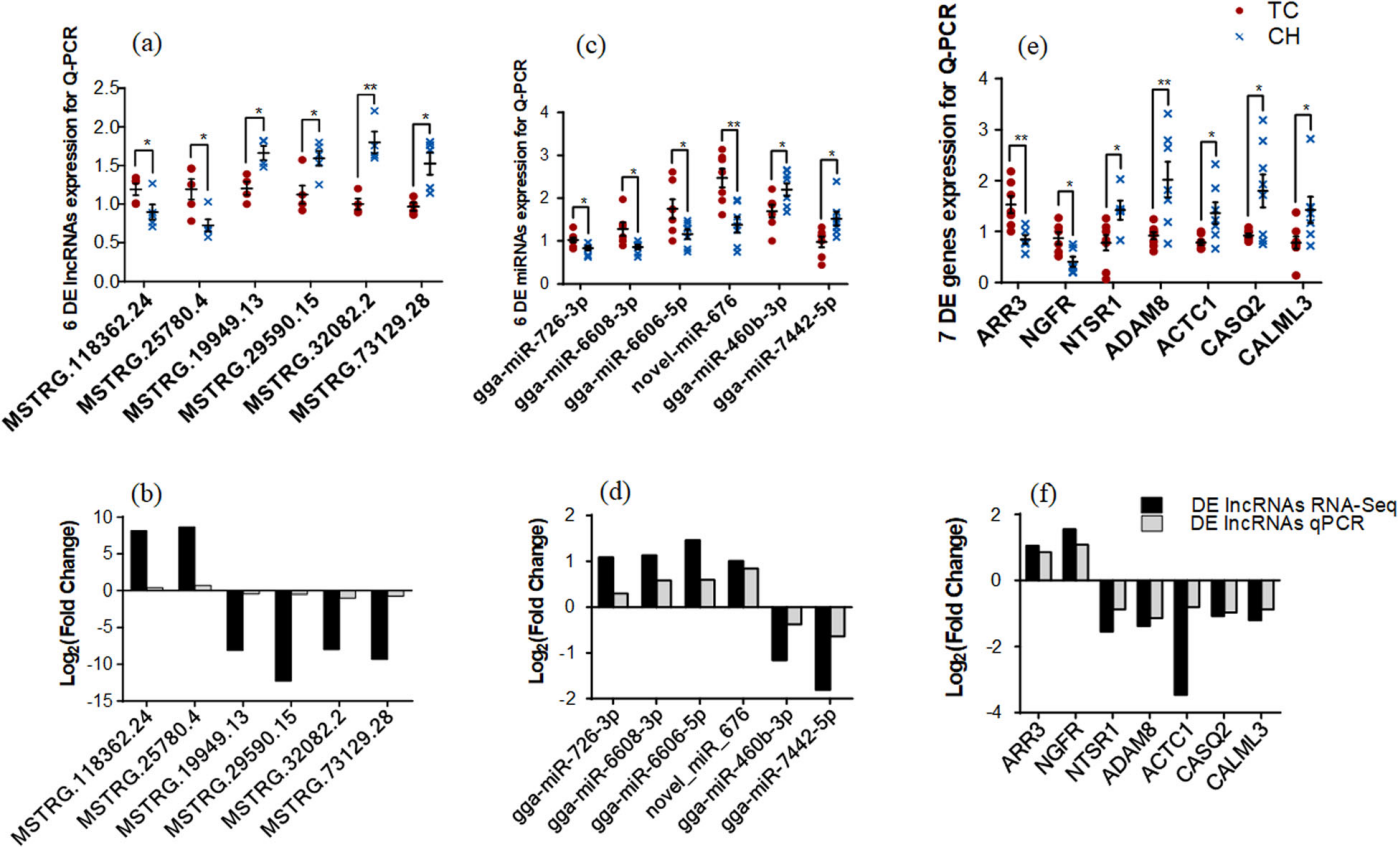
The expression of six differentially expressed (DE) lncRNAs (**a**), six DE miRNAs (**c**) and seven DE genes (**e**) validated with qRT-PCR in chicken embryo chorioallantoic membrane (CAM) tissue. Note: The Y-axis represents the expression value of RNA in chicken embryo CAM, and the X-axis represents the types of RNAs. Error bars represent SE of expression. * on the bars indicate *P* < 0.05 and ** indicate *P* < 0.01 between TC and CH breeds. Tibetan chickens (TCs, *n* = 4–8), and Chahua chickens (CHs, *n* = 4–8). The fold changes of the six DE lncRNAs (**b**), six DE miRNAs (**d**) and seven DE genes (**f**) showed that qRT-PCR results were consistent with the RNA-seq data

The expression levels of six DE lncRNAs (MSTRG.118362.24, MSTRG.25780.4, MSTRG.19949.13, MSTRG.29590.15, MSTRG.32082.2, and MSTRG.73129.28), six DE miRNAs (gga-miR-726-3p, gga-miR-6608-3p, gga-miR-6606-5p, novel-miR-676, gga-miR-460b-3p, and gga-miR-7442-5p), and seven DE genes (*ARR3*, *NGFR*, *NTSR1*, *ADAM8*, *ACTC1*, *CASQ2*, and *CALML3*) were measured using qRT-PCR to validate expression differences identified through RNA-seq. The results indicated that the expression levels of all RNAs were significantly different between TC and CH (*P* < 0.05), and fold-changes in expression followed the same trend in qRT-PCR and RNA-seq (Fig. 7).

## Discussion

Through evolution, Tibetan chickens have developed genetic adaptability to survive in low-oxygen environments. Previous reports have shown that Tibetan chickens exhibit unique phenotypic and physiological characteristics, including enhanced blood oxygen binding capacity and increased blood circulation [12, 17]. The ceRNA hypothesis was reported a few years ago and has now been widely accepted, which has improved our understanding of human diseases, as well as led to important advances in animal research [36, 37]. The recently discovered non-coding RNAs represent a new type of master regulator that affects gene expression and modulates a variety of cellular processes. Based on these theories, we constructed a ceRNA (lncRNA-miRNA-mRNA) regulatory network to clarify the molecular mechanism of Tibetan chicken embryo CAM adaptation to hypoxic environments.

The CAM, an important respiratory and circulatory organ for chicken embryo development, contains a large number of blood vessels, and hypoxia can induce increased blood vessel density [18, 38]. From the constructed ceRNA (lncRNA-miRNA-mRNA) network, we identified several miRNAs, including down-regulated novel-miR-589, novel-miR-815, novel-miR-85, novel-miR-669, as well as up-regulated miR-6606-5p, novel-miR-676, novel-miR-589, novel-miR-815, novel-miR-85, and novel-miR-567. Among these, miR-6606-5p was significantly up-regulated in the Tibetan chicken, and its target differentially expressed genes (*ACTC1*, *NCS1*, and *KCNMB4*) were found to be involved in vascular development under hypoxic conditions. *ACTC1* and *NCS1*, up-regulated in Tibetan chickens, were enriched in the GO term of blood circulation and regulated calcium ion transport;Ca^2+^ is necessary for the activity of *HIF-1*, which is a major transcriptional regulator of cells and development in response to hypoxia [39, 40]. *KCNMB4* has been reported to regulate blood pressure and was enriched in the GO terms of regulation of vasoconstriction, ion transport, and vascular smooth muscle contraction pathway [41]. Consequently, these findings indicate that Tibetan chickens can improve blood circulation and stimulate the activity of *HIF-1* through upregulation of *ACTC1* and *NCS1*, and Tibetan chickens promote vasodilation through downregulation of *KCNMB4* in CAM to adapt to hypoxic conditions. It is worth mentioning that with the exception of miR-6606-5p, other differentially expressed miRNAs, including miRNA-155, miRNA-302a, miR-302b-3p, miR-302b-5p, miR-302c-3p, miR-460b-3p, and miR-460b-5p, also play important roles in angiogenesis under hypoxia. The miRNA-155 promotes *HIF-1α* activity during prolonged hypoxia and participates in the PI3K/AKT pathway [42, 43]. Previous studies have shown that the miR-302 family (miR-302a/b/c/d) suppresses the proliferation, migration, and angiogenesis of vascular endothelial cells by targeting *VEGFA* [44–46]. The miR-460b-3p and miR-460b-5p were identified in animal models of hypoxic pulmonary hypertension and were involved in the regulation of *HIF-1α* [47, 48]. Moreover, other differentially expressed genes (*NGFR*, *ADAM8*, *CASQ2*, *IRF4*, *PTPRZ1*, *CALML3*, *ERBB4*, *ARR3*, and *NTSR1*) were enriched in blood vessel development, angiogenesis, blood circulation, hematopoiesis, response to hypoxia, oxygen transport, vascular smooth muscle contraction, calcium, and MAPK signaling pathways. The regulatory mechanism of these genes in hypoxic adaptation of Tibetan chickens is the same as that of *ACTC1*, *NCS1* and *KCNMB4* genes. In general, they regulate angiogenesis through the up or down-regulation of genes in their pathways. For example, *NGFR* is the receptor of *NGF*, which can induce chick CAM neovascularization [49]. Studies have shown that *ADAM8* is significantly induced by hypoxia and plays a role in the proliferation and migration of endothelial cells during angiogenesis [50, 51]. The explicit regulatory mechanism of these genes in hypoxic adaption of Tibetan chickens needs to be further studied. Therefore, we speculate that the enhanced tolerance of Tibetan chicken embryo CAM under hypoxic conditions is ascribed to miRNA-mediated modulation of the related target mRNAs, further regulating *HIF*, which enables Tibetan chicken to maintain hypoxic adaptation via the promotion of angiogenesis and blood circulation.

Glucose uptake and carbohydrate metabolism are the basis for the maintenance of normal physiological functions in humans and animals. Under hypoxic conditions, oxygen and carbon dioxide metabolism mainly depend on mitochondrial respiration and make use of adenosine triphosphate (ATP). Recently, a number of studies have shown the key regulatory role of energy metabolism, including glucose, carbohydrate, and lipid metabolic processes, among others, during hypoxic adaptation [52–55]. In the current research, from the constructed ceRNA network, we identified seven differentially expressed miRNAs (novel-miR-819, novel-miR-676, novel-miR-85, novel-miR-693, novel-miR-775, novel-miR-669, and novel-miR-867) and sixteen differentially expressed genes related to energy metabolism including glycosylation, glucose metabolism process, carbohydrate metabolism process, fatty acid metabolism, and ATP binding. For example, *SSTR5* and *NR1H4* are essential for glucose homeostasis and play a pivotal role in glucose metabolism in animals [56, 57]. Cortisol regulates the metabolism of mouse adipose cells through the serotonin receptor gene *HTR2C*, and genetic variation of the *APOA1* gene is linked to lipid metabolism and cardiovascular disease risk [58, 59]. It should be considered that more differentially expressed genes (*KCNB1*, *P3H2*, *CHST8*, *LYZ*, *HAO2*, *ACER1*, *ACSBG1*, *ELOVL2*, *ELOVL3*, *GBE*, and *NOX3*) are targeted by key miRNAs, and several studies have shown that these mRNAs are also involved in energy metabolism. Therefore, we speculate that Tibetan chickens have enhanced energy metabolism due to the function of these RNAs, allowing for adaptation to hypoxic conditions.

Accumulating evidence indicates that lncRNAs play the role of ceRNAs (or miRNA sponges) in a variety of biological processes, including high-altitude adaptation. Such as polymorphisms of LINC-PINT and LINC00599 are associated with high-altitude pulmonary edema in Chinese populations [60]. Another study reported the expression profiles of lncRNAs in mice with high-altitude hypoxia-induced brain injury and provided new insights into the molecular mechanism of its treatment [61]. In addition, a previous study reported the expression profiles of lncRNAs responsible for fatness and fatty acid composition traits in Tibetan pigs [62]. In the current work, we found that the aforementioned differentially expressed genes involved in angiogenesis and energy metabolism were targeted by 37 differentially expressed lncRNAs in the ceRNA network, suggesting that these lncRNAs may also function as miRNA sponges and may play a role in the hypoxic adaptation of Tibetan chicken embryos with regard to angiogenesis and energy metabolism.

**Fig. 8:**
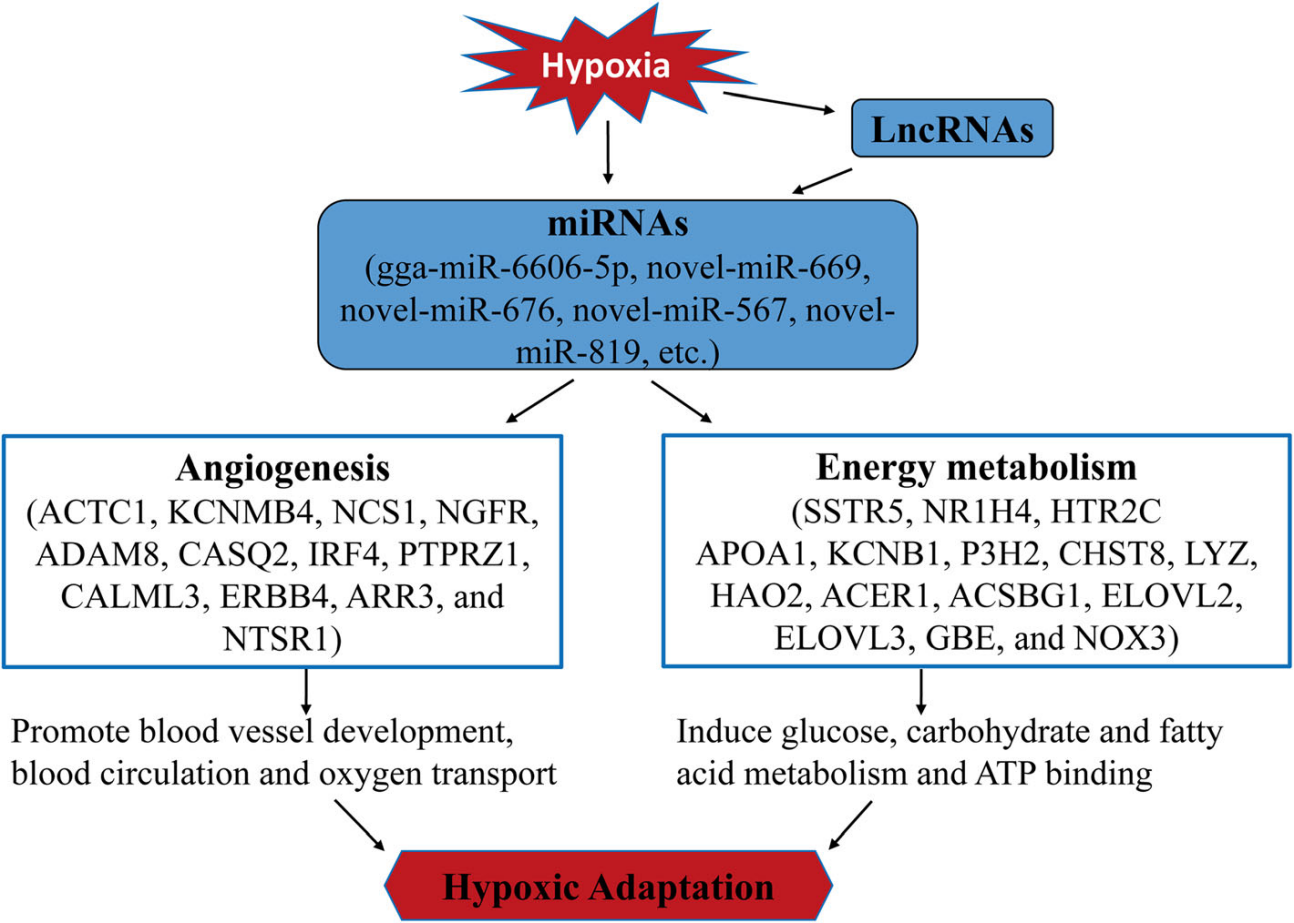
Proposed model of hypoxic adaptation in Tibetan chicken embryos. Under hypoxic conditions, miRNAs act as key regulators that directly target important differentially expressed (DE) genes. In this process, some lncRNAs can act as ceRNAs to competitively bind the miRNA response element (MRE) of miRNAs, indirectly affecting the expression of mRNAs

Based on the ceRNA theory and the ceRNA network constructed in this study, we propose a mode of action of differentially expressed lncRNAs, miRNAs, and mRNAs during hypoxic adaptation of Tibetan chicken embryos (Fig. 8). Under hypoxic conditions, miRNAs act as key regulators to modulate the up or down-regulation of important differentially expressed genes. As a consequence, the angiogenesis/blood circulation of chorioallantoic capillaries and energy metabolism, such as glucose/carbohydrate metabolism, are stimulated, leading to enhanced hypoxia adaptability of Tibetan chicken embryos. In this process, some lncRNAs act as ceRNAs to competitively bind the miRNA response element of miRNAs, which may indirectly affect the expression of mRNA.

## Conclusions

In conclusion, 389 DE lncRNAs, 73 DE miRNAs, and 354 DE genes were identified between Tibetan and Chahua chickens. The transcriptomic data revealed several key candidate ceRNAs (DE lncRNAs-DE miRNAs-DE genes) that may play high-priority roles in the hypoxic adaptation of Tibetan chickens by regulating angiogenesis and energy metabolism. These results provide insights into the molecular mechanisms of hypoxic adaptation regulatory networks from the perspective of coding and non-coding RNAs.

## Supplementary Information

**Additional file 1: Table S1.** The qRT-PCR primers of six DE lncRNAs, six DE miRNAs and seven DE genes for identification by RNA-seq.

**Additional file 2: Table S2.** List of software and the parameters used in this study.

**Additional file 3: Table S3.** Summary of sequencing reads aligned with the *Gallus gallus* genome.

**Additional file 4: Figure S1.** Lengths of mRNAs and lncRNAs distribution. **Figure S2.** Figure of hierarchical clustering of 389 DE lncRNAs (a), 73 DE miRNAs (b), and 354 DE genes (c) in Tibetan and Chahua chickens. **Figure S3.** Comparative analysis of exon and ORF lengths of lncRNAs and mRNAs. **Figure S4.** Distribution of the DE lncRNAs and DE mRNAs on the different chromosomes. **Figure S5.** Network of ceRNA (lncRNA-miRNA-mRNA) including 162 DE lncRNAs, 25 DE miRNAs, and 108 DE genes.

**Additional file 5: Table S4.** The 3194 known lncRNAs, 471 known miRNAs, and 968 novel miRNAs.

**Additional file 6: Table S5.** The 389 differentially expressed lncRNAs (DE lncRNAs), 73 DE miRNAs, and 354 DE genes.

**Additional file 7: Table S6.** GO and KEGG pathway enrichment of differentially expressed lncRNA (DE lncRNA) target genes.

**Additional file 8: Table S7.** GO and KEGG pathway enrichment of differentially expressed miRNA (DE miRNA) target genes.

**Additional file 9: Table S8.** GO and KEGG enrichment of differentially expressed mRNAs (DE genes).

**Additional file 10: Table S9.** Important difference in circRNA-miRNA-mRNA interactions based on the competing endogenous RNA (ceRNA) network.

## Data Availability

All RNA-seq data were deposited in the Gene Expression Omnibus under accession number GSE160324.

## Abbreviations

lncRNAs: Long non-coding RNA
miRNA: MicroRNA
TC: Tibetan chicken
CH: Chahua chicken
CAM: Chorioallantoic membrane
DE: Differentially expressed gene
DE lncRNA: Differentially expressed lncRNA
DE miRNA: Differentially expressed miRNA
ceRNA: Competing endogenous RNA
GO: Gene Ontology
KEGG: Kyoto Encyclopedia of Genes and Genomes
qRT-PCR: Quantitative real-time PCR
ORF: Open reading frame
