# Supplementary material for: Comprehensive analysis of coding and non-coding RNA transcriptomes related to hypoxic adaptation in Tibetan chickens
Source: J Anim Sci Biotechnol. 2021 May 3;12:60. doi: 10.1186/s40104-021-00582-2 (PMC8091548; doi:10.1186/s40104-021-00582-2)
Supplement: Supplementary file 4 — Additional file 4: Figure S1. Lengths of mRNAs and lncRNAs distribution. Figure S2. Figure of hierarchical clustering of 389 DE lncRNAs (a), 73 DE miRNAs (b), and 354 DE genes (c) in Tibetan and Chahua chickens. Figure S3. Comparative analysis of exon and ORF lengths of lncRNAs and mRNAs. Figure S4. Distribution of the DE lncRNAs and DE mRNAs on the different chromosomes. Figure S5. Network of ceRNA (lncRNA-miRNA-mRNA) including 162 DE lncRNAs, 25 DE miRNAs, and 108 DE genes. [file 40104_2021_582_MOESM4_ESM.pdf]

Figure S1

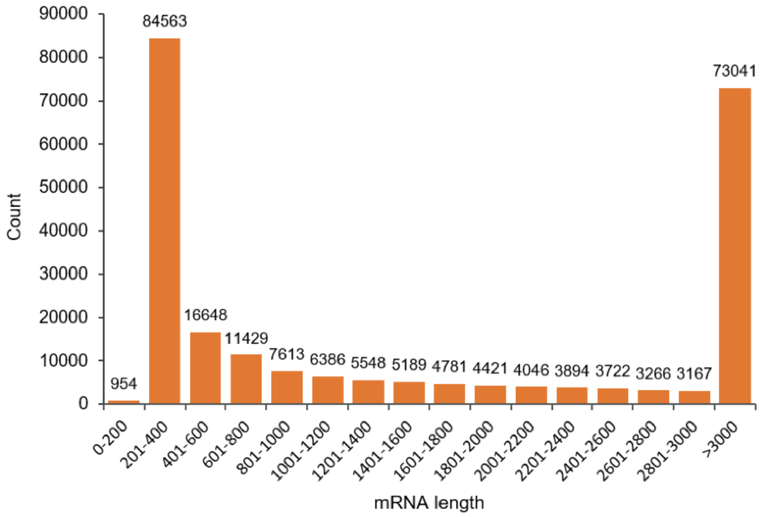

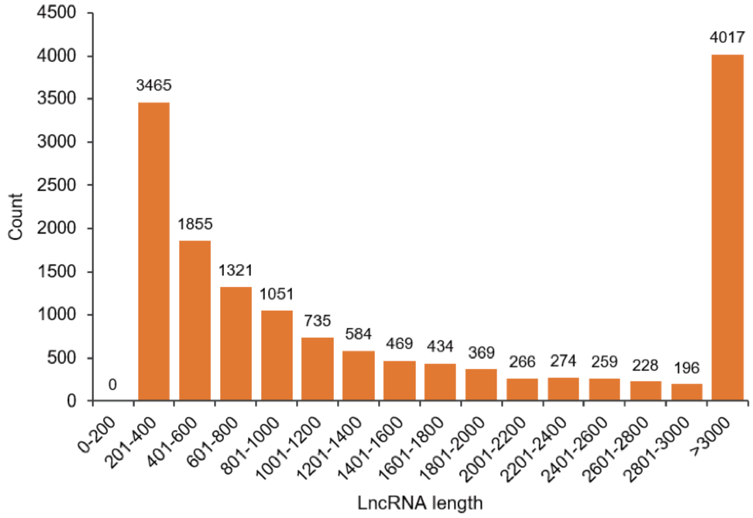

Fig. S1. Lengths of mRNAs and lncRNAs distribution. The abscissa is the length (bp), and the ordinate is the number of mRNAs and lncRNAs whose length is distributed within the range.

Figure S2

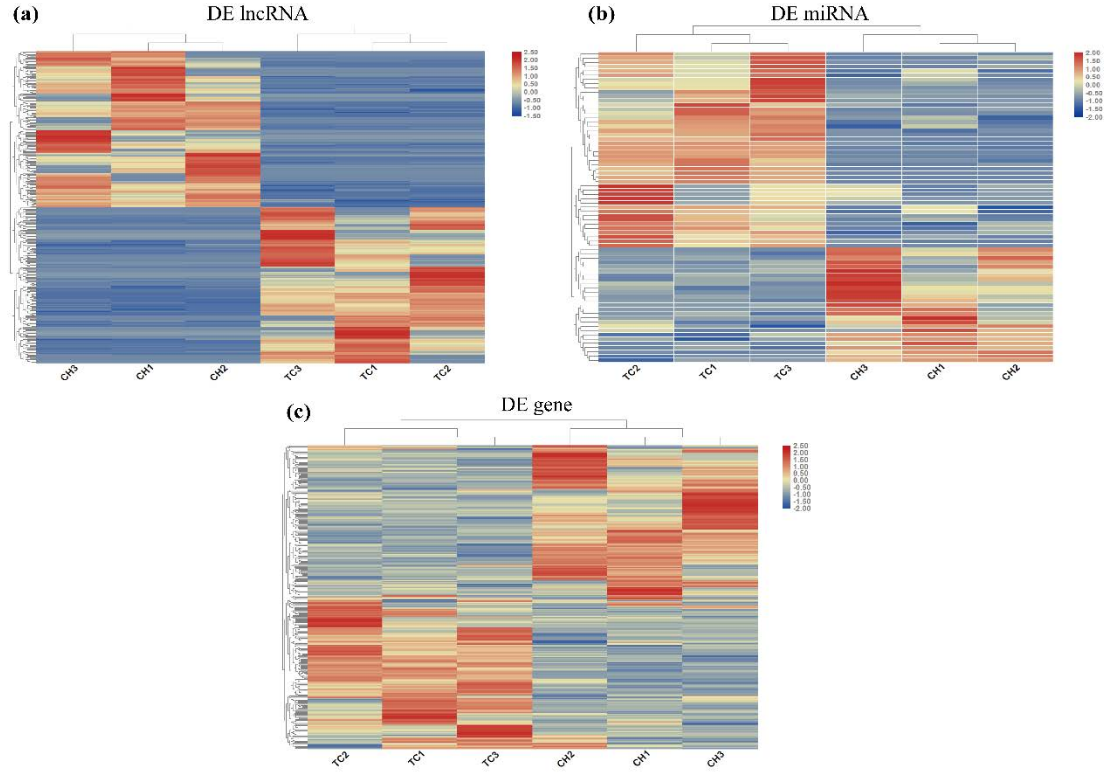

Fig. S2. Figure of hierarchical clustering of 389 differentially expressed (DE) lncRNAs (a), 73 DE miRNAs (b), and 354 DE genes (c) in Tibetan and Chahua chickens. X and Y coordinates represented samples and differentially expressed RNAs, respectively. Red shows high-expressed RNAs, blue shows low-expressed RNAs. Vertical comparison indicated that differentially expressed RNAs could be classified into two categories with opposite directional variation. TC was Tibetan chicken; CH was Chahua chicken.

Figure S3

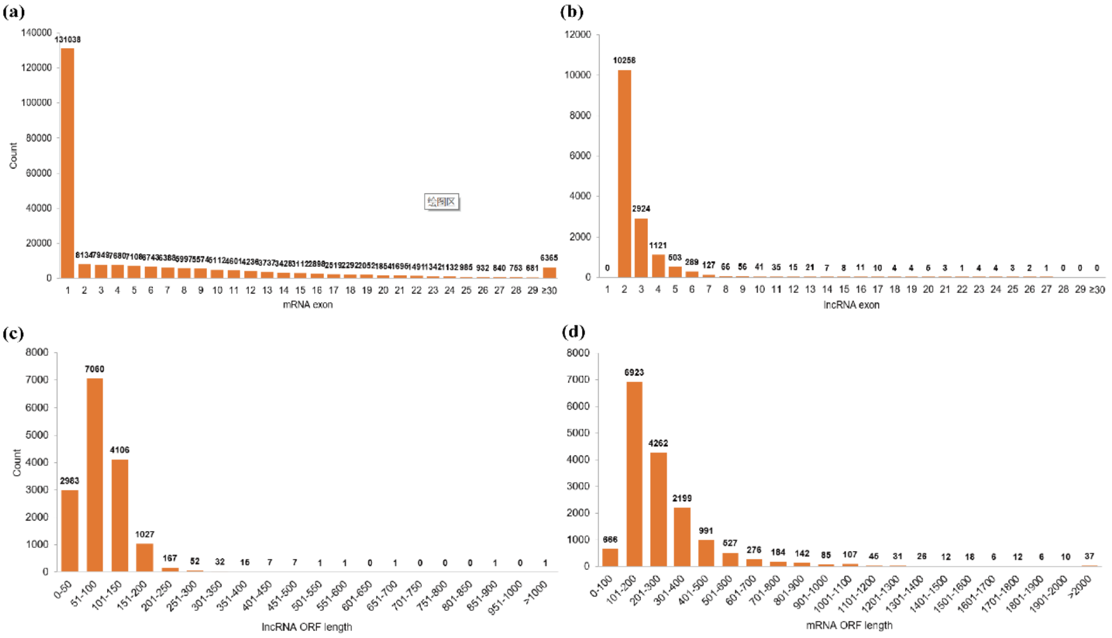

Fig. S3. Comparative analysis of exon and ORF lengths of lncRNAs and mRNAs. Statistics of exon number of lncRNA (a) and mRNA (b); the abscissa is the number of exons, and the ordinate is the number of lncRNAs/mRNAs with the number of exons distributed in the range. Statistics of ORF length of lncRNA (c) and mRNA (d); the abscissa is the ORF length, and the ordinate is the number of lncRNAs/mRNAs whose length is distributed in the range.

Figure S4

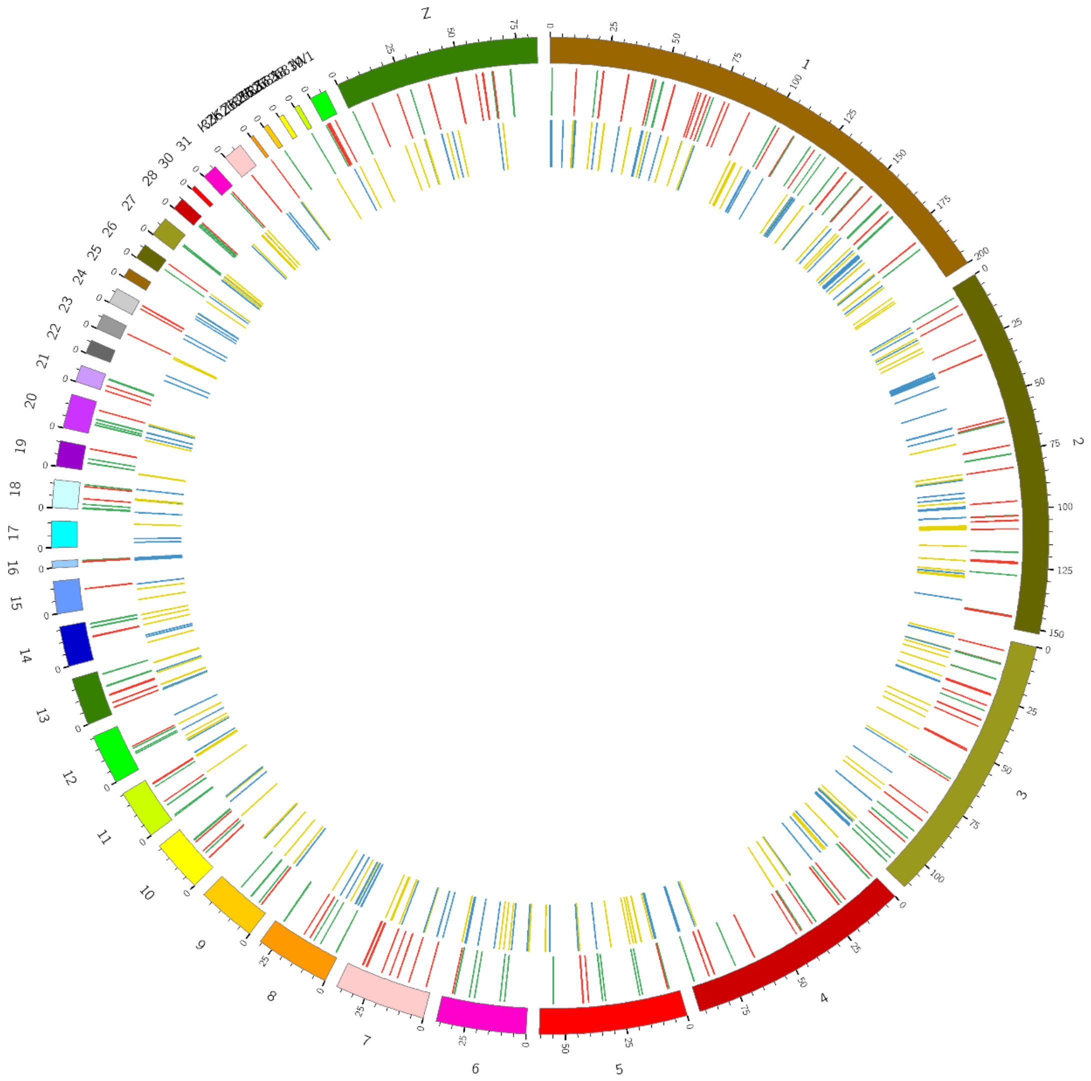

Fig. S4. Distribution of differentially expressed (DE) lncRNAs and DE mRNAs on chicken chromosomes. The outer ring is the chromosome of the chicken reference genome, the middle ring is the distribution of DE mRNAs on the chromosome, the inner ring is the distribution of DE lncRNAs on the chromosome, the red color represents up-regulated mRNAs, the green color represents down-regulated mRNAs, the yellow color represents up-regulated lncRNAs, and the blue color represents down-regulated lncRNAs.

Figure S5

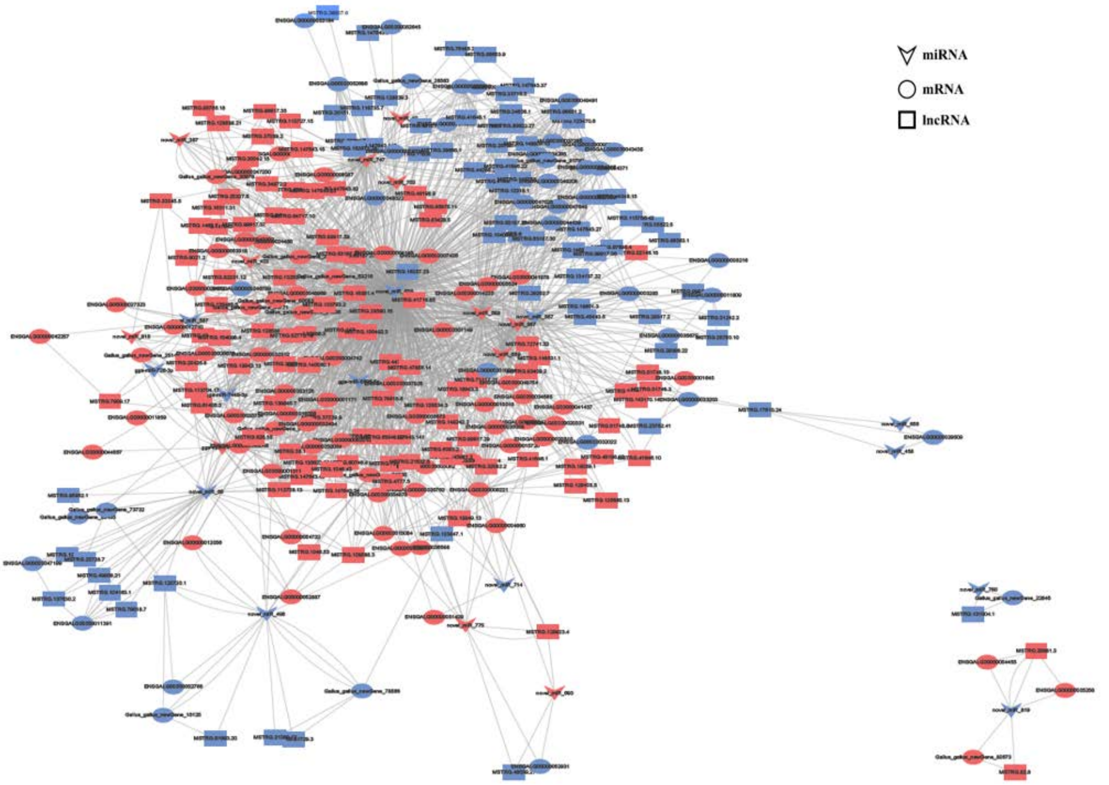

Fig. S5. Network of ceRNA (lncRNA-miRNA-mRNA) including 162 DE lncRNAs, 25 DE miRNAs and 108 DE genes. Color represents the up-regulated (red color) and down-regulated (blue color) levels.
